# It Is Time to Consider the Lost Battle of Microdamaged Piezo2 in the Context of *E. coli* and Early-Onset Colorectal Cancer

**DOI:** 10.3390/ijms26157160

**Published:** 2025-07-24

**Authors:** Balázs Sonkodi

**Affiliations:** 1 Department of Health Sciences and Sport Medicine, Hungarian University of Sports Science, 1123 Budapest, Hungary; bsonkodi@gmail.com; 2 Department of Sports Medicine, Semmelweis University, 1122 Budapest, Hungary; 3 Faculty of Health Sciences, Institute of Physiotherapy and Sport Science, University of Pécs, 7624 Pécs, Hungary; 4 Physical Activity Research Group, Szentágothai Research Centre, University of Pécs, 7624 Pécs, Hungary

**Keywords:** early onset colorectal cancer, *Escherichia coli*, colibactin, Piezo2, circadian regulation, hippocampal ultradian clock, proton

## Abstract

The recent identification of early-onset mutational signatures with geographic variations by Diaz-Gay et al. is a significant finding, since early-onset colorectal cancer has emerged as an alarming public health challenge in the past two decades, and the pathomechanism remains unclear. Environmental risk factors, including lifestyle and diet, are highly suspected. The identification of colibactin from *Escherichia coli* as a potential pathogenic source is a major step forward in addressing this public health challenge. Therefore, the following opinion manuscript aims to outline the likely onset of the pathomechanism and the critical role of acquired Piezo2 channelopathy in early-onset colorectal cancer, which skews proton availability and proton motive force regulation toward *E. coli* within the microbiota–host symbiotic relationship. In addition, the colibactin produced by the *pks* island of *E. coli* induces host DNA damage, which likely interacts at the level of Wnt signaling with Piezo2 channelopathy-induced pathological remodeling. This transcriptional dysregulation eventually leads to tumorigenesis of colorectal cancer. Mechanotransduction converts external physical cues to inner chemical and biological ones. Correspondingly, the proposed quantum mechanical free-energy-stimulated ultrafast proton-coupled tunneling, initiated by Piezo2, seems to be the principal and essential underlying novel oscillatory signaling that could be lost in colorectal cancer onset. Hence, Piezo2 channelopathy not only contributes to cancer initiation and impaired circadian regulation, including the proposed hippocampal ultradian clock, but also to proliferation and metastasis.

## 1. Introduction

The recent identification of early-onset mutational signatures with geographic variations by Diaz-Gay et al. is a significant finding, since early-onset colorectal cancer (_eo_CRC) has emerged as an alarming public health challenge in the past two decades, and the pathomechanism remains unclear [1]. Environmental risk factors, including lifestyle and diet are highly suspected [1,2]. The identification of colibactin of *Escherichia coli* (*E. coli*) as a potential pathogenic source [1] is a major step forward in addressing this very recent public health challenge.

In support, an earlier finding showed an important interrelation, namely, there is a clear age dependence in the microbial features between _eo_CRC (<50 years) and late-onset colorectal cancer (_lo_CRC) (>65 years) [3]. Moreover, the microbial–host link was stronger in _eo_CRC, implicating a more direct association with tumorigenesis through an unidentified cancer-related pathomechanism in contrast to _lo_CRC [3]. Notably, age-dependent epidemiological variations in colorectal cancer (CRC) are likely associated with differences in environmental factors, rather than genetic predisposition [4,5]. These factors involve lifestyle exposures, such as dietary intake, physical activity, alcohol consumption, obesity, and disruptions in the circadian rhythms in the _eo_CRC-affected younger age-group [6].

The following opinion manuscript aims to outline the Piezo2 ion channel-dependent neurocentric onset or the neurocentric gateway to pathophysiology in the aforementioned mysterious pathomechanism of CRC due to acquired Piezo2 channelopathy, especially in the case of _eo_CRC. It is important to note that the principality of Piezo2 is not only presented in proprioception, as demonstrated by the team of Nobel laureate Ardem Patapoutian [7], but is also suggested to be present in its microdamage, as the abovementioned principal gateway to pathophysiology, potentially even leading to cancer [8]. Notably, this neurocentric opinion piece is fully in line with emerging observations that the nervous system is actively involved not only in invasion and metastasis but also in the initiation of CRC [9].

## 2. Piezo2 Channelopathy, Dysbiosis, and Circadian Regulation

**Figure 1:**
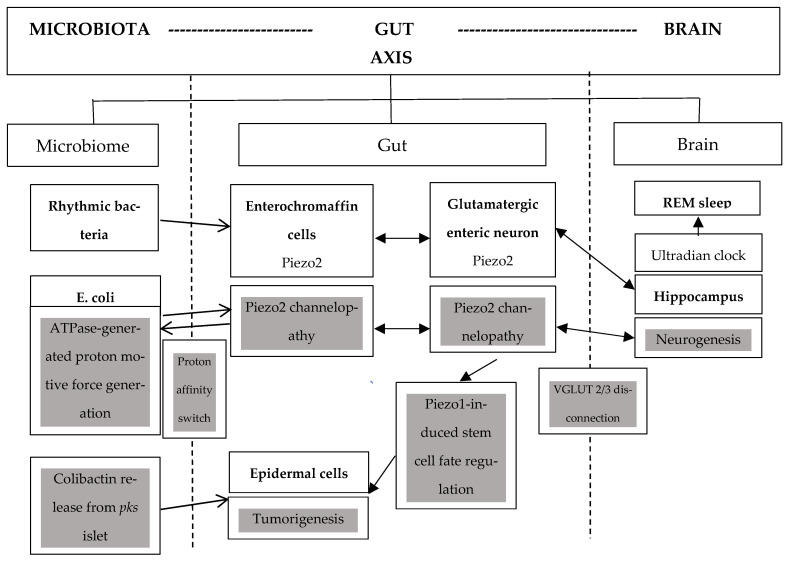
The novel ultrafast proton-based oscillatory synchronization mechanism from rhythmic bacteria to hippocampus, constructing the ultradian backbone of the microbiota–gut–brain axis. The grey boxes denote the proton affinity switch induced Piezo2 channelopathy, VGLUT2/3 disconnection, and colibactin-derived tumorigenesis entailing miswired pathway in colorectal cancer.

A recent paper theorizes that there is an unaccounted underlying quantum-tunneled ultrafast long-range proton-coupled oscillatory synchronizational pathway to the hippocampus from the enterochromaffin cells (ECs), e.g., from the colon and rectum [10]. Moreover, this manuscript also proposes that the intact microbiota–gut–brain axis is likely accountable for a novel synchronizational mechanism towards ultradian and circadian regulation from rhythmic bacteria of the microbiota to hippocampal memory formation [10]. The ultrafast signaling regulation of the ultradian rhythm may be initiated by activated Piezo2-induced proton motive force, derived from mitochondrial oxidative phosphorylation (OXPHOS), with the involvement of VGLUT3 through allosteric transmission at a distance (Figure 1) [10]. Notably, no other proprioceptive ion channel, other than Piezo2, could initiate this quantum mechanical free-energy-stimulated ultrafast concerted proton tunneling [10]. Hence, the acquired channelopathy of Piezo2 at ECs and nerve terminals of the microbiota–gut–brain axis may pose a critical impairment (Figure 1) that is suggested to be one gateway to dysbiosis [10]. It is important to note that lifestyle and dietary factors influence the circadian clock, and circadian rhythms vanish in human CRC, as patient-derived organoid-based research shows [2]. Additionally, lifestyle factors, such as obesity, sedentary behavior, and microbiome diversity changes with age, and antibiotic use all have Piezo relevance. In support, emerging research highlights the important role of Piezo2 in adipose sensory innervation [11]; these Piezo2-containing sensory neurons also regulate systemic and adipose tissue metabolism [12]. Not only excessive overstimulation but also prolonged understimulation of Piezo2 posits a risk, as part of a sedentary lifestyle, through the inability to properly initiate the ultrafast long-range proton-based signaling within the nervous system. The lack of this ultrafast signaling initiation also leads to miswiring within the nervous system [13]. Moreover, Piezo2-containing cells unfortunately decrease with age [14], as Piezo2 function declines due to repeated channelopathy and protein degradation in an age-dependent manner [8,15]. This age-dependent degradation of Piezo2 function [8,15] contributes to the age-dependent microbiome diversity changes, as explained hereafter. Finally, antibiotic use is associated not only with _eo_CRC but also with _lo_CRC [16]. Doxycycline, a broad-spectrum antibiotic, is even used in animal tumor research in order to suppress inducible PIEZO1 shRNA expression that in return suppresses tumor growth and increases survival, hence revealing the essential role of Piezo1 in tumor growth [17].

In support of the underlying ultrafast oscillatory synchronizational pathway of the microbiota–gut–brain axis, some of the gut bacteria within the microbiota exhibit rhythmic oscillations in synchrony with the circadian clock [18,19]. They are coined as rhythmic bacteria, incorporating approximately 10–15% of gut microbial bacteria [18,19]. Recently, it was hypothesized that these rhythmic bacteria contribute to dysbiosis at disease onset [20]. Correspondingly, circadian-related diseases were analyzed in respect to this link, such as type 2 diabetes, hypertension, atherosclerotic cardiovascular diseases, inflammatory bowel disease, metabolic syndrome, and most importantly CRC [20]. In fact, a relationship was detected between rhythmic bacteria and circadian-related diseases, although this association was weak [20]. Conclusively, these rhythmic bacteria are part of a more complex pathophysiology at disease onset when it comes to circadian disruptions [20]. Part of this complexity is that these rhythmic bacteria are a vast minority among the most abundant bacteria, reflecting that not only does circadian disruption affect them, but other physiological stressors do as well [20]. Of note, confounding lifestyle variables of CRC are all Piezo ion channel-related functionally, as depicted above, while chronic stress is known to induce dysbiosis and CRC cancer growth [21,22]. Consequently, chronic stress also promotes CRC progression by increasing beta-catenin expression [21]. Moreover, Piezo-mediated responses are significantly altered because they are biochemically and functionally tethered to the actin cytoskeleton through the cadherin–beta–catenin mechanotransduction complex [23]. Accordingly, chronic stress induces dysbiosis, while Piezo channelopathy decreases rhythmicity and increases stiffness in the affected given microenvironment in favor of CRC cancer growth, as an underlying mechanism. Thereby, the author of this manuscript proposes that the above findings are highly in line with the acquired Piezo2 channelopathy-induced disrupted VGLUT3 signaling along the microbiota–gut–brain axis [10], impaired ultradian rhythmicity, and stress regulation. In addition, this Piezo2 channelopathy-induced microscopically undetectable VGLUT disconnection will lead to the abovementioned switch or miswiring within the nervous system [8].

Furthermore, the earlier mentioned paper also theorized that Piezo2-containing ECs interact with oscillatory serotoninergic enteric neurons within the enteric nervous system, and this pathway initiates a slower circadian rhythm domain of the gut–brain axis towards circadian regulation [10]. Moreover, the aforementioned ultradian excitatory glutamatergic ultrafast proton-based signaling modulates rapid eye movement (REM) sleep, while the latter serotoninergic one modulates non-REM sleep, respectively [10].

Even more importantly, the implicated circadian clock disruptions trigger transformation by driving *APC* loss of heterozygosity, leading to Wnt signaling hyperactivation [2]. It is noteworthy that chronic Piezo2 channelpathy-induced switch/miswiring is proposed to result in a state of “part of wound healing kept alive” [24], leading to chronic pathological remodeling [25] with the involvement of Wnt signaling [26]. Hence, the onset of CRC pathophysiology is a gateway to pathological remodeling or a derailed wound healing process.

## 3. Colibactin, *Escherichia coli*, and Piezo2

Diaz-Gay et al. also associated colibactin exposure with *APC* driver mutations in _eo_CRC [1]. In support, not only may the impaired protective function of microbiome count in dysbiosis, but the resultant increase in cancer promotion may too [3]. Correspondingly, microbial signaling also affects the host by a gene expression-coupled mechanism [27], leading to microbiota-induced genomic instability in CRC [28]. The current author proposes that this genomic instability is the result of acquired Piezo2 channelopathy due to its principle transcription activator feature [29].

Colibactin is the genotoxic metabolite product of *E. coli*, produced by its polyketide synthase *(pks)* island [30]. In addition, *E. coli* has the feature of regulating proton motive force, depending on bacterial growth phases, in order to sustain cell energy balance during fermentation regardless of various carbon sources [31]. Acute Piezo2 channelopathy is suggested to be transient non-contact microdamage with an underlying proton affinity switch. However, the repeated bout effect of this non-contact microdamage without adequate regeneration over a period of time could chronify this proton affinity switch, the equivalent of chronic Piezo2 channelopathy [8]. This chronic state could skew the proton availability and proton motive force regulation toward *E. coli* within the microbiota due to the fermentative energy-limited conditions (Figure 1). This will lead eventually to impaired homeostatic energy balance equilibrium within the symbiotic microbiota–host interaction, the equivalent of dysbiosis. Therefore, under this chronic energy scarcity, the Piezo2-containing ECs and somatosensory neurons are competitively disadvantaged, and a vicious circle may prevail. This dysregulated process leads to accelerated aging that includes cancer development by uncontrolled growth, depending on environmental risk factors and genetic predisposition [8]. Polyketide synthase-positive (*pks^+^*) *E. coli* has been shown to be a central player in this pathomechanism [1].

However, the exact function of *pks* island and the genetic aspect of colibactin-induced tumorigenesis is far from entirely known, for example, the transfer of colibactin from *E. coli* to host cells is not completely understood [30], although, it has been known for a while that the expression of these *pks* island genes is highly correlated with the presence of a carbon source [32]. In particular, glucose has the ability to enhance these gene expressions [32]. The toxic effect of colibactin induces DNA damage by exploiting the Wnt proteins of the host cells [33]. The current authors suggest that this colibactin-induced Wnt signaling-involved DNA damage could conflict with the aforementioned Piezo2 channelopathy-induced pathological remodeling with Wnt signaling involvement (Figure 1). Hence, it presents a break in the proper wound healing process, leading to sustained pathological remodeling and transcriptional dysregulation. Moreover, *pks^+^ E. coli* causes damage, leading to mutations and genomic instability and promotes carcinogenesis and tumor development [30,34,35,36].

In support, *E. coli* strains under certain growth stages are prone to proton motive force generation [31]. This buildup of proton motive force, depending on the growth stage of *E. coli*, could cause a proton affinity switch in the Piezo2 content of EC cells, leading to acquired Piezo2 channelopathy. Moreover, Piezo1 is demonstrated to contribute to force-induced ATP secretion [37]. The current author suggests an analogous ATP secretion mechanism of Piezo2 as well. In addition it is worth considering from the ATP secretion aspect that a Piezo2–Piezo1 crosstalk may exist in a given fluid-filled compartment with a selective barrier, such as the gut [10]. Correspondingly, Piezo activation-induced ATP efflux may serve the ATPase of *E. coli* for proton motive force generation. Hence, the switch in the growth phase of *E. coli* could result in the upregulation of ATP, producing OXPHOS in EC cells and attached Piezo2-containing excitatory glutamatergic neurons as well. However, OXPHOS will be depleted over time as a direct result of the proton affinity switch, leading to a metabolic switch of Piezo2-containing ECs and glutamatergic neurons. This is analogous to the DOMS mechanism, where it is also theorized that the depletion of OXPHOS results in a neural metabolic switch [38].

However, the upregulated OXPHOS promotes cell proliferation and tumorigenesis in CRC, e.g., through the Prohibitin 2—NADH:ubiquinone oxidoreductase core subunit S1 pathway mediation [39]. This is why the dual inhibition of OXPHOS and glycolysis has a synergistic antitumor effect in CRC [40]. Nevertheless, this dual inhibition is not due to preventing a metabolic switch, because, according to the current author, the metabolic switch is already an underlying factor in CRC due to Piezo2 channelopathy. Accordingly, dual inhibition of OXPHOS and glycolysis shifts the metabolic loading to a lower energy generation pathway among parallel metabolic pathways. Hence, this strategy does not metabolically feed the Piezo2-microdamaged and -switched rapid (not ultrafast anymore) glutamatergic VGLUT3-containing neural signaling. Therefore, proton affinity switch could switch mitochondrial energy metabolism to mitochondrial glucose and glutamine fermentation pathways from the evolutionarily superior energy-generating OXPHOS and glutamine respiration pathways [38]. In support, this glucose and glutamine respirofermentation could run simultaneously in fast growth micromilieu, like in cancer [41].

An interesting proposition is the imprinting of SBS88 and ID18 during upbringing on the epithelium of the colon in the presence of *pks^+^* bacteria, leading to the loss or gain of these bacteria decades later [1]. An important consideration is that prolonged excessive stretch/distention or chemical destruction may microdamage Piezo2 [8], and this may have relevance in the colon and rectum as well. Consequently, acquired Piezo2 channelopathy-induced simultaneous transcription activation with the aforementioned memory dimensions and later-in-life dysbiosis may explain the proposition of Diaz-Gay et al.

In support, Piezo2 is remarkably elevated in CRC [42]. The current author translates this phenomenon as a feedforward compensatory upregulation due to chronic Piezo2 channelopathy. Unfortunately, this increased Piezo2 presence was shown to promote proliferation and metastasis of CRC, beyond its role in the occurrence and development of this cancer type [42]. The ‘how’ will be explained in the subsequent section. For the time being, it should be emphasized that the hippocampus is not only the prime location for learning and memory but for adult hippocampal neurogenesis as well. Therefore, the ‘switched’ or ‘miswired’ secondary compensatory signaling along the underlying ultradian ECs–hippocampal axis may explain the promotion of proliferation [38] and metastasis in CRC. Furthermore, Piezo1 activation should be contemplated in neighboring cells within the compartmental micromilieu due to the impaired/lost Piezo2–Piezo1 crosstalk [8]. Indeed, mechanical forces through Piezo1 signaling have a role within gastrointestinal tumors when it comes to tumor growth and metastasis [43]. Doxycycline administration also showed in another animal tumor research model that Piezo1 is essential for tumor growth [17].

Another factor to consider is that syndecan-2 is upregulated both in CRC [44] and in the acute inflammation of the colon [45] as well. In both cases, acquired Piezo2 channelopathy is suspected as the initiating pathophysiology [10]. Since syndecans are suspected as the critical first-line player of the Piezo2–Piezo1 crosstalk, and Piezo2 channelopathy impairs this crosstalk [25], the current author proposes that syndecan-2 upregulation should be viewed as a feedforward modulation by acquired Piezo2 channelopathy. Syndecan expression is enigmatic in a given cell, tissue, and developmental stage. Accordingly, every cell exhibits at least one syndecan out of the four members of the syndecan family. Hence, syndecan-2 is specific to CRC pathomechanism due to its role in cell adhesion, motility, proliferation, and differentiation [44]. The negative charge of syndecans is viewed as important in proton collection; hence, the shedding of these proteoglycans likely alters the electrostatic micromilieu in the given microenvironment, leading to Piezo2 channelopathy [25]. Indeed, shed syndecan-2 promotes tumorigenesis in CRC cells [46] and cancer progression [47], which the Piezo2 channelopathy theory or the gateway to pathophysiology in CRC initiation.

## 4. Blue Light Link to Colorectal Cancer

The current author puts forward that excessive overloading of the gut–eye axis and its link to the microbiota should be regarded as related to the _eo_CRC pathomechanism. Notably, Piezo2 is also present on the cornea and retina and likely initiates the ultrafast proton-based signaling as the underlying backbone of the eye–brain axis, where the hippocampus is the integrative hub [8,10]. Photoreceptors in the eye, called retinal ganglion cells (RGCs) [48], are highly sensitive to blue light, and they directly communicate with the brain [49], including the hippocampus [50]. These RGCs contain Piezo2 and Piezo1, not to mention their role in RGC damage [51] with the involvement of the aforementioned Wnt/beta-catenin signaling pathway [52]. Hence, the integration of the ultradian backbone of the eye–brain axis and the microbiota–gut–brain axis through the hippocampal hub would explain the so-called gut–eye axis. Visible light is synchronized to our inner clock of the suprachiasmatic nuclei of the hippothalamus within the daily 24 h cycle [49]. Short wavelength blue light, out of the visible light spectrum, is the strongest contribution to circadian system synchronization [49]. One study showed that 30 min exposure to blue light one hour prior to bedtime in healthy youngsters had the impact of a 30-min phase shift delay in the onset of REM sleep [53]. It is also noteworthy that Piezo2 initiated the ultrafast proton-based cross-frequency coupled oscillatory synchronizational signaling mechanism, as the ultradian domain of circadian regulation may construct the ultradian clock in the hippocampus [10]. Furthermore, this Piezo2-initiated ultradian rhythm from the gut to the hippocampus, as the ultrafast ultradian backbone of the gut–brain axis, is proposed to modulate REM sleep [10]. Interestingly, VGLUT1 and VGLUT2 are present in RGCs with distinct functions [54], as they are implicated in the Piezo2-initiated ultrafast proton-based allosteric signaling at a distance to the hippocampus [8]. In particular, VGLUT2 and melanopsin expression in subsets of RGCs seems to carry relevance in the VGLUT2-signaled pathway in the non-image forming function of the retina [54] and circadian regulation. Moreover, VGLUT3 is present in amacrine cells that provide glutamatergic input into RGCs through motion and contrast activation [55]. In addition, these amacrine cells selectively convey the blue-ON signal and may provide the blue-OFF signal [56]. Piezo2-initiated co-functioning of Piezo2 and ASIC2 is theorized according to the ultrafast proton-based signaling via VGLUT2 [25]. As an analogy, Piezo2-initiated co-functioning of Piezo2 with RGCs and amacrine ASIC1a may be the case in the proton-based signaling through VGLUT3. In support, ASIC1a is present in both of these cells [57]. Therefore, the aforementioned integration of the eye–brain axis and the microbiota–gut–brain axis through the hippocampal hub would explain the so-called gut–eye axis through Piezo2-initiated VGLUT2 and VGLUT3 allosteric transmission at a distance. Indeed, subcellular pathways through VGLUT3-expressing amacrine cells are detected that serve the object–motion-selective signals in the retina [58].

Additionally, over-excessive intense light exposure induces pathological alterations in the retina, including the RGCs [59]. These alterations involve the disruption of intracellular REDOX and Ca^2+^ homeostasis, endoplasmic reticulum stress, and inflammation, leading to irreversible retinal damage [59]. Of note, the development of CRC also involves REDOX imbalance [60], disrupted Ca^2+^ homeostasis, endoplasmic reticulum stress [61], and inflammation [62]. The role of evolutionarily conserved Piezo in the buffering of endoplasmic reticulum stress through Ca^2+^ handling is known [63], and Piezo2 is theorized to modulate the ultradian oscillator Hes1 gene expression through reactive oxygen species (ROS) [10]. Notably, ROS regulate Hes1 [64]. The oscillatory expression of Hes1 is modulated by the transcriptional negative feedback loop with delayed timing; hence, Hes1 represses its own promoter [64,65,66]. However, it is known that this negative feedback loop with shorter delays diminishes oscillations but makes them faster [67]. Therefore, the current author proposes that 30 min of ultradian sensory activation of Piezo2 right before sleep induced by blue light result in the 30-min phase shift delay at night with the involvement of Hes1 activation, when it should not happen. Moreover, prolonged over-excessive blue light exposure prior to sleep could induce Piezo2 channelopathy, resulting in the dysregulated modulation of Hes1 and switch or miswiring due to a disrupted VGLUT2 and VGLUT3 signaling pathway.

Accordingly, the author of this paper does not find it to be accidental that blue light exposure before sleeping has a detrimental effect on the circadian regulation [49]. Therefore, the rapidly increasing exposure to blue light-emitting devices right before sleep in the past two decades may also contribute to the degradation of the gut–eye axis and increased incidence of _eo_CRC. Resultant Piezo2 channelopathy-induced transcription activation [8] and dysregulated Hes1 activation [10] could be the reason why Hes1 contributes to cell proliferation, differentiation, and migration in CRC [68]; it also promotes CRC progression [69], invasiveness [70], and even chemoresistance [71]. Furthermore, it explains ‘how’ the chronic Piezo2 channelopathy-induced increase in PIEZO2 promotes the occurrence, development, proliferation, and metastasis of CRC. In addition, the co-functioning of Piezo2 and ASIC2 in the proposed ultrafast proton-based signaling via VGLUT2 [25] also explains why ASIC2 is overexpressed in CRC [72] in a feedforward manner, induced by Piezo2 channelopathy. Finally, the abovementioned co-functioning of Piezo2 and ASIC1a in the proposed ultrafast proton-based signaling via VGLUT3 explains not only the function of hippocampal learning and memory but also emotional and cardiovascular regulation [73]. Moreover, the colocalization of hippocampal VGLUT3 and the inhibitory GABA [73] explains the aforementioned blue-OFF signal pathway along the bidirectional ultrafast proton-signaled ultradian backbone of the eye–brain axis. Conclusively, chronic acquired Piezo2 channelopathy ultimately could not only lead to pathological remodeling or unfinished wound healing, ongoing dysregulated transcription activation [8], dysbiosis, dysautonomia [10], and impaired blue-OFF signal but also explain the emotional dimension of the known psychological stress impact on CRC [22] and known autonomic dysregulation in CRC [74].

## 5. Nerve Growth Factor and Colorectal Cancer

In line with the current neurocentric CRC tumorigenesis opinion concept, recent research shows that neuro-mesenchymal crosstalk modulated by the β2 adrenergic-nerve growth factor feedforward loop promotes the progression of CRC [75]. This is a significant finding, because of the scientific debate of what comes first in regard to primary damage, where one side demonstrated that increased nerve growth factor (NGF) initiates the pathophysiology onset [76]. However, the other side of the debate claims that transient Piezo2 channelopathy-induced sensory miswiring or switch may contribute to NGF production by mesenchymal cells, as a result of miswired neural crosstalk [38]. Accordingly, NGF production could be the result of sensory terminal Piezo2 channelopathy-derived switched signaling and impaired cross-frequency coupling of Piezo2–Piezo2 and Piezo2–Piezo1 and, consequently, impaired Piezo1-driven cell orientation and adjustment, not to mention transcription activation [38]. Notably, Piezo2 channelopathy-induced impaired Piezo2–Piezo2 crosstalk may come with autonomic dysregulation [8,38] and increased sympathetic loading [77]. Again, autonomic dysregulation is present in CRC patients where the sympathetic and parasympathetic imbalance leads to sympathetic overdrive [74]. Moreover, it is known that Piezo1 contributes to remodeling through fibroblasts [78] by continuously adapting to the changes in matrix stiffness [79]. Accordingly, the current author translates the significant findings of Kobayashi et al. [75] as follows: Piezo2 channelopathy-induced impaired Piezo2–Piezo2 crosstalk will increase the sympathetic loading and resultant β2 adrenergic-derived NGF secretion from cancer-associated fibroblasts. These cancer-associated fibroblasts are activated by Piezo2 channelopathy induced impaired Piezo2–Piezo1 crosstalk in the given tumor microenvironment in the aforementioned Piezo1-dependent feedforward manner [17].

Additionally, NGF follows ultradian variations in the plasma levels of healthy people in a light- and sleep-dependent fashion [80]; however, even shorter-term rhythms of episodic ultradian events should be considered as well. In support, a forgotten study showed earlier that temporal correlation could exist between the heart rate, medullary units (important autonomic regulation locus in the brain), and hippocampal theta rhythm, both awake and during sleep [81]. Moreover, the same author showed that even visual and auditory discharges could engage into phase-locked temporal correlation with the hippocampal theta rhythm, as part of ultradian brain rhythms [82]. Interestingly, it was also detected that a temperature-sensitive ultradian rhythm exists in fibroblasts, independent of the cell cycle and circadian clock [83]. Significant traumatic brain injury research showed the essential contribution of Piezo2 in the stress-related defensive arousal response (DAR) [84]. DAR is an essential survival mechanism, and it is turned on by perceived threat and evoked by visual and auditory inputs in relation to motor abilities [84]. The author of this manuscript proposes that these findings are analogous to the proposed underlying Piezo2-initiated ultrafast ultradian underlying brain axes, synchronized to the hippocampal theta rhythm [8,10]. Correspondingly, acquired Piezo2 channelopathy impairs this ultrafast ultradian synchronization in CRC, resulting in autonomic dysregulation, increased sympathetic loading, and impaired ultradian regulation of NGF. This initiating Piezo2 channelopathy results in elevated NGF release in the short-term, like in _eo_CRC and _lo_CRC, but depletion in the long-term due to chronification of Piezo2 channelopathy and aging. In support, NGF, as essential for neural survival, growth, and maintenance, indeed shows a decline in an age-dependent manner [85]

## 6. Conclusions

The current opinion manuscript is in appreciation of the significant finding of Diaz-Gay et al. and aims to promote this future angle of science and research in order to confront the rising tendency of _eo_CRC in the past two decades. Mechanotransduction converts external physical cues to inner chemical and biological ones, although not without limits. Correspondingly, the quantum-tunneled ultrafast long-range proton-coupled oscillatory synchronizational pathway to the hippocampus, initiated by Piezo2, seems to be the principal and essential underlying novel neurocentric signaling that is likely lost at CRC onset. Moreover, this primary damage not only contributes to CRC tumorigenesis but also to impaired circadian regulation, tumor cell proliferation, and metastasis. Future work should focus on testing this neurocentric CRC initiation pathway by the use of 3D organoids, animal models, in vitro Piezo2 disruption studies, mathematical and computational modeling, further analyzation of rhythmic and other interacting bacterial contents of microbiota, and the ultradian–circadian regulation by precision polysomnography and heart rate variability analysis.

## Data Availability

Not applicable.
